# How to interpret the results of clinical studies: “Multicenter cutting‐edge study” or “real‐world big data study”?

**DOI:** 10.1002/ags3.12681

**Published:** 2023-04-27

**Authors:** Yoshihiro Nabeya

**Affiliations:** ^1^ Division of Esophago‐Gastrointestinal Surgery Chiba Cancer Center Chiba Japan

**Keywords:** big data study, cutting‐edge, multicenter study, real‐world

While a prospective randomized controlled trial would be the best way to prove a hypothesis scientifically in a clinical study, it is often difficult to design in practice and it usually takes a long time to come to a conclusion. Therefore, even in retrospective studies, it is desirable to draw conclusions from a proactive perspective and to propose decision‐making for improving clinical outcomes. However, single‐institutional study conduct, small sample size, and lack of adjustment for background factors have been identified as limitations in retrospective clinical studies. In order to solve such drawbacks of retrospective studies, “*multicenter*” joint studies in which plural institutions provide data and studies using “*big data*” such as Japanese Diagnosis Procedure Combination (DPC) database or National Clinical Database (NCD) with already inputted clinical information have often been conducted in recent years. In this issue, novel findings from four “*multicenter*” studies and four “*big data*” studies are reported.

A multicenter study is suitable for examining subjects with a small number at a single institution. Umeda et al. have proposed the appropriate extent of lymphadenectomy in surgery for intrahepatic cholangiocarcinoma according to tumor location, by a multicenter data analysis of the therapeutic index of 279 patients (DOI:10.1002/ags3.12631).^1^ By a multicenter retrospective cohort study of 1,623 patients followed by propensity score matching (PSM), Nakatsutsumi et al. have revealed that the prophylactic use of negative‐pressure wound therapy with delayed sutures was associated with a lower incidence of incisional surgical site infection after emergency laparotomy for colorectal perforation.^2^ If the number of subjects is large, PSM can be used to adjust background factors and obtain reliable results with scientifically valid analyses. Noda et al. have demonstrated that neoadjuvant chemotherapy for resectable colorectal liver metastasis might prolong the recurrence‐free survival period in 192 patients with intermediate risk of postoperative recurrence by data analysis of 20 institutions with PSM (DOI:10.1002/ags3.12643). Ikeda et al. analyzed quality of life (QOL) data after proximal gastrectomy, utilizing a central registration system of the Postgastretomy Syndrome Assessment Scale (PGSAS)‐45 questionnaires, which were distributed to eligible patients in multiple institutions. This study has revealed that the QOL of double tract reconstruction (172 cases) was relatively superior to that of esophagogastrostomy reconstruction (300 cases) in patients with remnant stomach sizes of around 1/2 and 2/3 (DOI:10.1002/ags3.12645).

In “*multicenter*” studies, even with a large number of cases or in a prospective fashion, there may be bias in the selection of participating institutions. A multicenter study conducted by only high‐volume centers may provide especially “*cutting‐edge*” evidence, but it may be sometimes difficult to apply the novel knowledge to clinical practice universally at any facility. The results of multicenter studies should be introduced carefully to clinical practice with this important point in mind. However, the results of four multicenter studies published in this issue is expected to be introduced generally to daily clinical practice.

By extracting and examining the necessary information from “*big data*” of the DPC database, which is known as Japan's nationwide administrative database, we can truly see the current “*real‐world*” situation of the topic. Using the DPC database on 941 patients undergoing surgery for congenital biliary dilatation (CBD) at 357 hospitals, Mori et al. have demonstrated that laparoscopic surgery (177 patients) for CBD had short‐term results comparable to those of open surgery (764 patients), while high cost of laparoscopic surgery is an issue that requires further research to solve (DOI:10.1002/ags3.12630).^3^ By using DPC data and analyzing through PSM, Oba et al. compared the efficacy and safety of mechanical bowel preparation (MBP, 1756 cases) alone and MBP combined with oral antibiotic bowel preparation (MOABP, 1756 cases) for rectal cancer surgery (DOI:10.1002/ags3.12641).^4^ While there has been a trend to simplify preoperative preparation before colorectal surgery due to the prevalence of enhanced recovery after surgery program, this study has shown the “*real‐world*” information that MOABP for rectal surgery was associated with a decreased incidence of postoperative complications without increasing the incidence of *Clostridium difficile* colitis and methicillin‐resistant *Staphylococcus aureus* colitis (DOI:10.1002/ags3.12641).^4^ Maeda et al. have reported the impact of the COVID‐19 pandemic on the number of gastrointestinal surgeries in Japan in 2020, utilizing data of 228,860 cases from NCD, demonstrating a marked reduction in surgeries for gastric and rectal cancers with early T factors that may reflect prioritization of surgeries and reduction in cancer screenings (DOI:10.1002/ags3.12638).^5^ This paper has also highlighted that the quality of surgery was maintained in terms of reduced mortalities and morbidities in Japan, while the long‐term effects of this pandemic should be monitored (DOI:10.1002/ags3.12638).^5^ By using NCD data, Kajiwara et al. have reported the number of gastroenterological surgical procedures and short‐term results over the 10‐year period up to 2020 (DOI:10.1002/ags3.12662). These clinically important facts or trends cannot be clarified without “*big data*.”

For advancement of gastroenterological surgery, both “*cutting‐edge*” evidence and “*real‐world*” information are necessary. The results of “*multicenter*” studies should be interpreted in light of the number of participating institutions and the volume of hospitals and surgeons, which affect universality of the results. We should always understand this point and introduce the “*cutting‐edge*” evidence according to the current situation at each facility. On the other hand, the results of “*big data*” analyses may be regarded as “*real‐world*” information of the topic, the reports of which have recently increased in the field of clinical surgery. However, the DPC database or NCD based on medical practices in clinical settings have been expected to improve the quality of medical care and economy, and the information can be limited in scope and accuracy. Therefore, it may be necessary to add and organize survey items with the accumulation of data with future research in mind.

Finally, this issue also includes other excellent original articles and informative review articles. I hope you will introduce the novel knowledge obtained from these papers to your surgical practice and research activities from tomorrow.

## CONFLICT OF INTEREST STATEMENT

Yoshihiro Nabeya is a current Associate Editor of *Annals of Gastroenterological Surgery* and declares no other conflicts of interests for this article.

## ETHICS STATEMENT

Approval of the research protocol: N/A.

Informed Consent: N/A.

Registry and the Registration No. of the study/trial: N/A.

Animal Studies: N/A.
